# Field Emission Characteristics of the Structure of Vertically Aligned Carbon Nanotube Bundles

**DOI:** 10.1186/s11671-015-1005-1

**Published:** 2015-07-17

**Authors:** Pao-Hung Lin, Cong-Lin Sie, Ching-An Chen, Hsuan-Chen Chang, Yi-Ting Shih, Hsin-Yueh Chang, Wei-Jhih Su, Kuei-Yi Lee

**Affiliations:** Department of Electronic and Computer Engineering, National Taiwan University of Science and Technology, No. 43, Sec. 4, Keelung Road, Taipei, 10607 Taiwan; Graduate Institute of Electro-Optical Engineering, National Taiwan University of Science and Technology, No. 43, Sec. 4, Keelung Road, Taipei, 10607 Taiwan

**Keywords:** Vertically aligned carbon nanotube (VACNT) bundles, Arrangement, Thermal chemical vapor deposition (CVD), Field emission (FE)

## Abstract

In this study, we performed thermal chemical vapor deposition for growing vertically aligned carbon nanotube (VACNT) bundles for a field emitter and applied photolithography for defining the arrangement pattern to simultaneously compare square and hexagonal arrangements by using two ratios of the interbundle distance to the bundle height (*R*) of field emitters. The hexagon arrangement with *R* = 2 had the lowest turn-on electric field (*E*_to_) and highest enhancement factor, whereas the square arrangement with *R* = 3 had the most stable field emission (FE) characteristic. The number density can reveal the correlation to the lowest *E*_to_ and highest enhancement factor more effectively than can the *R* or *L*. The fluorescent images of the synthesized VACNT bundles manifested the uniformity of FE currents. The results of our study indicate the feasibility of applying the VACNT field emitter arrangement to achieve optimal FE performance.

## Background

Since its discovery in 1991, carbon nanotube (CNT) [1] properties have been explored and examined, and several applications have been designed and developed. The properties of CNT, namely a low work function (*Φ*), high aspect ratio, excellent electrical property, and mechanical stiffness, make it one of the promising field emission (FE) materials. The performance of FE materials determined on the basis of the Fowler–Nordheim (FN) equation [2], which describes the relation between the FE current number density, *Φ*, and enhancement factor *β*, is influenced by the nature of the material and the arrangement and surface morphology of the field emitter. Being used to determine and control the factors of FN equation, many approaches have improved the FE performance of CNTs, such as the appropriate alignment of CNTs [3], geometrical arrangement [4], film morphology and CNT bundle number density [5], FE stabilization [6], long lifespan [7], and uniformness [8].

Because the materials used for various emitter fabrications are identical, the arrangement of an emitter decisively influences FE characteristics, primarily, the screening effect and the number density of the emission site. The relation between the arrangement and FE characteristics has been widely studied, and the CNT has been used as an emitter. Nilsson and coworkers [9] have reported that when the CNT had a height and radius of 1 and 2 nm, respectively, and the number density was in the range of 10^7^ cm^−2^, the ratio of approximately twice the inter-CNT distance to the height of CNT effectively concentrated the applied electric field, enabling reaching the maximum FE current density. In addition, by using three-dimensional simulations, Smith et al. [8] demonstrated that an equilibrium is required between the screening effect and the number of emission sites for an emitter to perform effectively. A maximum FE efficiency was predicted to be achieved if the distance between the neighboring emitters compared with an emitter’s height was threefold. In addition to investigating emitter interdistance and height, Hong et al. demonstrated that a hexagonal field emitter has a relatively lower field screening effect than does a square field emitter [10]. Nevertheless, when an overconcentrated electric field was applied to the field emitter for generating a high FE current, the heat produced during the FE destroyed the emitter. Therefore, balancing the arrangement and architecture factors to obtain the maximum CNT emitter FE efficiency is essential. Moreover, the formations of such arranged individual CNTs are too complex to process perfectly, and the prior treatment of catalyst metal nanoparticles for individual CNT growth is costly. Previous studies have indicated that such a desired architecture consisting of individual vertically aligned CNTs (VACNTs) could be replaced by VACNT bundles [11]. When a VACNT bundle contains a high number density of CNTs, the screen effect causes the CNT bundle to approximate an isolated CNT emitter. The pattern of CNT bundle arrays can be defined easily by using photolithography; the required catalyst metal can then be deposited on the defined position. This method is simple, easy to control, and inexpensive.

In this study, the design patterns of CNT bundles (hexagonal and square arrangements) were defined using photolithography. The VACNT bundles were synthesized by thermal chemical vapor deposition (CVD). The growth time was adjusted to control the CNT height. The ratios of the length (*L*) of the neighboring CNTs to their height (*H*) were set as two and three. When the ratio of *L* to *H* was set as two or three, the number density of CNT bundles was confined in a range to determine the number density of the emission site. In addition to the basic measurement of the current density versus the applied electric field (*J*–*E*), a long-term test and the uniformity of the fluorescent screen were examined to display the FE characteristics. Uniformity in the hexagonal arrangement is expected to be more satisfactory than that in the square arrangement considering the edge effect [8].

## Methods

The VACNT bundles were synthesized using thermal CVD on a Si substrate with an area of 10 mm × 10 mm. The Si substrates were ultrasonically cleaned with ethanol before CNT synthesis. On the Si substrates in square and hexagonal configurations, circular patterns with a 10-μm diameter and two pitches (30 and 45 μm) were defined using photolithography. A 5-nm Al buffer layer and a 3-nm catalytic Fe film were then deposited on the substrates by using electron beam evaporation. C_2_H_2_ gas was subsequently introduced as a carbon source in the thermal CVD system with a working pressure of 4 Torr at 750 °C. The height (*H*) of synthesized VACNT bundles was controlled by the growth time.

This study examined four types of configurations. Table 1 lists the sample codes (A, B, C, and D) that correspond to the arrangements, *L*, *H*, and the ratio *R*. The length *L* was defined to be the distance between two adjacent centers of the circles. The four samples exhibited two types of arrangements (square and hexagonal) formed with two ratios, namely *R* = 2 and 3.

**Table 1:** Arrangement, interbundle distance, bundle height, and ratio of interbundle distance to bundle height

Sample	Arrangement	Interbundle distance (μm)	Bundle height (μm)	Ratio of interbundle distance to bundle height
		(*L*)	(*H*)	(*R*)
A	Square	30	15	2
B	Square	45	15	3
C	Hexagon	30	15	2
D	Hexagon	45	15	3

We also investigated the surface morphologies of the synthesized VACNT bundles by using a scanning electron microscope (SEM). The FE properties of the CNT bundle arrays were examined using a high-vacuum system including a pair of parallel plate electrodes with 20-μm diameter configuration under a pressure of about 5 × 10^−7^ Torr. A SourceMeter (Keithley 2410) supplied the applied voltage, and the FE current was measured. The gap between the anode (stainless steel) and cathode (sample) was 150 μm. The FE measurement was for an array of emitters, that is, the entire area of the synthesized CNT bundle arrays; accordingly, the current density is the FE current divided by the sample area. The sample area was 10 mm × 10 mm. To examine the distribution uniformity of the electrons emitted from the emitter, a fluorescent screen experiment was carried out in another high-vacuum chamber with an observation window on a wall.

## Results and Discussion

**Fig. 1:**
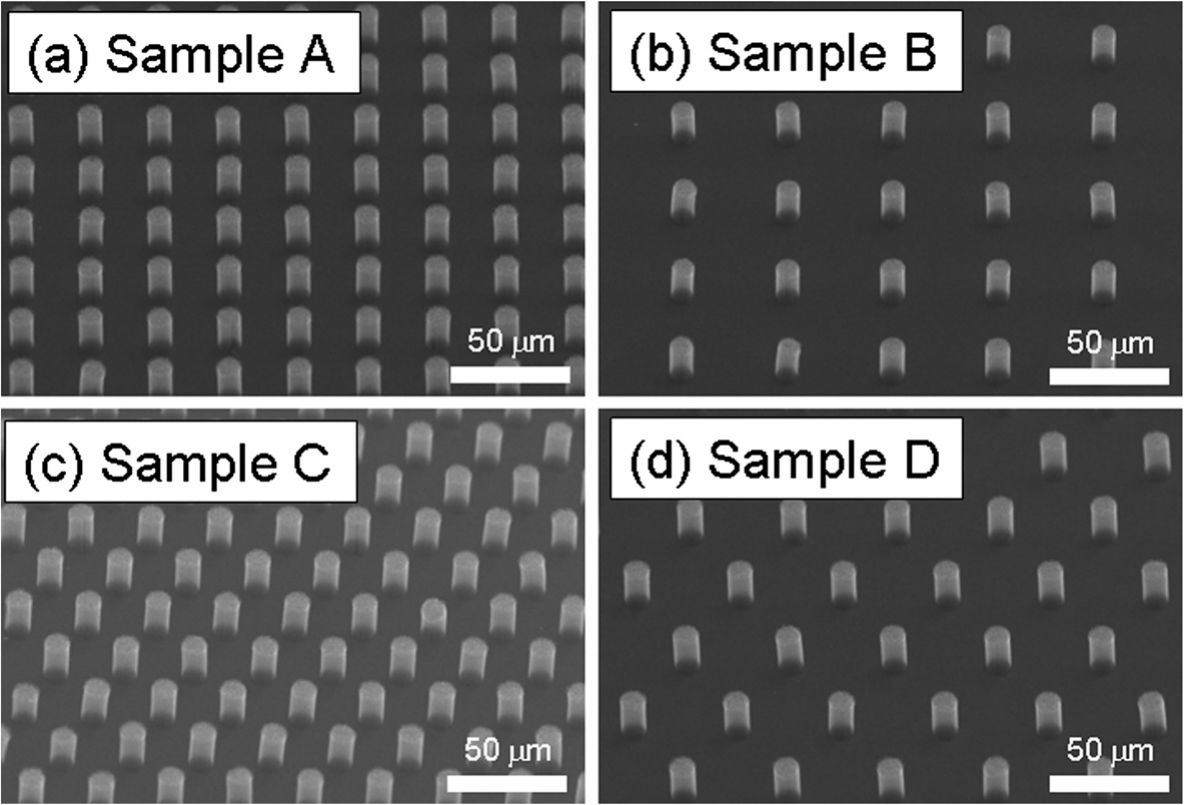
SEM images of VACNT bundles of different arrangements. **a** Square, *R* = 2. **b** Square, *R* = 3. **c** Hexagon, *R* = 2. **d** Hexagon, *R* = 3

Figure 1 shows the tilted SEM images of the synthesized VACNT bundles. Each synthesis of CNT bundles was grown regularly according to the designed mask pattern. The arrangement patterns of samples A and B were square, and samples C and D were hexagonal. The lengths of samples A and C were 30 μm and those of samples B and D were 45 μm, and the calculated number densities of the VACNT bundles of samples A, B, C, and D were 1.0 × 10^5^, 4.9 × 10^4^, 1.6 × 10^5^, and 7.0 × 10^4^ cm^−2^, respectively.

**Fig. 2:**
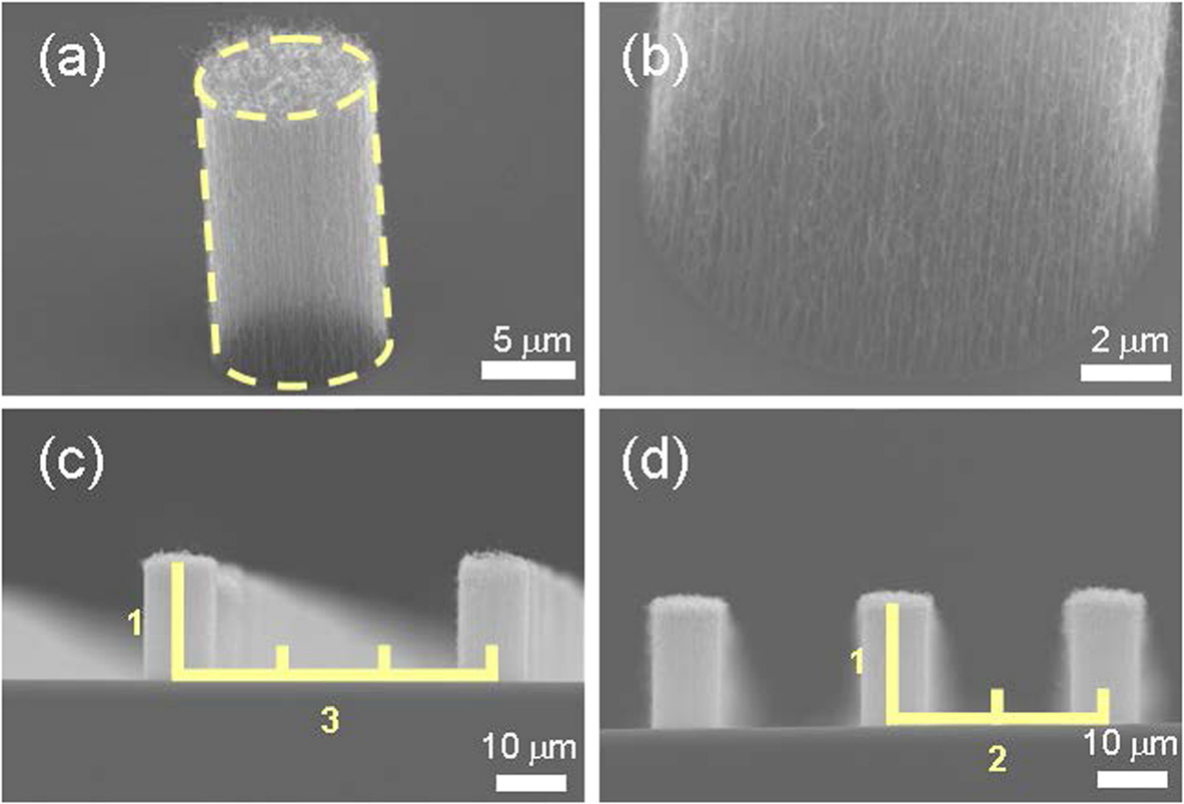
SEM images of VACNT bundles. **a** An isolated cylindrical CNT bundle. **b** The bottom of the CNT bundle. **c** CNT bundles arranged in an interval three times their height. **d** CNT bundles arranged in an interval two times their height

Figure 2a shows the SEM image of a single VACNT bundle. The shape of the VACNT bundle was cylindrical, and the diameter and height were 10 and 15 μm, respectively. Figure 2b shows an enlarged SEM image of Fig. 2a. The bundle was vertically aligned with the Si substrate; it consisted of VACNTs with a high number density of approximately 10^9^ cm^−2^, which was examined using the magnified SEM image of the bottom region. The applied electric field varied acutely in the peripheral edge of the VACNT bundle because of a screening effect. Consequently, the VACNT bundle could be almost treated as an isolated emitter [12]. Moreover, because the height of the VACNT bundles was fixed as 15 μm, according to the designed pattern, the ratios of *L* to *H* were three and two, as shown in Fig. 2c, d, respectively.

**Fig. 3:**
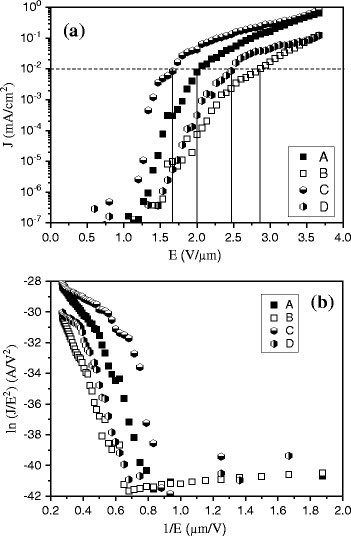
Field emission characteristics of samples *A*, *B*, *C*, and *D*. **a** *J*–*E* plot and **b** FN plot

Figure 3a presents the *J*–*E* curves of the synthesized VACNT bundles. The turn-on electric fields (*E*_to_) corresponding to the current density of 10^−2^ mA cm^−2^ [13, 14] for samples A, B, C, and D were approximately 2.0, 2.8, 1.6, and 2.5 V μm^−1^, respectively. Figure 3b shows the FN plots of the synthesized samples. The *β* of each sample was calculated as $\mathrm{slope}=-0.434\left(\frac{B{\varPhi}^{3/2}}{\beta}\right)$, where $B=4\sqrt{2m}\frac{q^{1/2}}{3\hslash }=6.83\times {10}^9\left(\frac{{\mathrm{V}\ \mathrm{m}}^{-1}}{{\left(\mathrm{eV}\right)}^{3/2}}\right)$, which was derived by the log of the FN equation, $J=\left(\frac{q^2{\beta}^2{E}^2}{16{\pi}^2\hslash \varPhi}\right) \exp \left[\frac{-4\sqrt{2m}{\left(q\varPhi \right)}^{3/2}}{3\mathit{\hslash q\beta E}}\right]$, where *J* is the FE current density (A cm^−2^), *E* is the applied electric field (V cm^−1^), *q* is the charge (Coulomb), *ħ* is Planck’s constant divided by 2*π*, and *m* is the electron mass. When the *Φ* of CNT was set at 4.8 eV [15], the enhancement factors (*β*) of samples A, B, C, and D were 1020, 840, 1770, and 905, respectively. Table 2 shows a summary of the relation among the *E*_to_, *β*, and number density of VACNT bundle samples. Evidently, *E*_to_ decreased as *β* and the VACNT bundle number density increased. The correlation between the number density and *E*_to_, or the correlation between the number density and *β*, reveals that the number density is a more favorable indication compared with the ratio of *R* or *L*. According to the FE results, the ratio *R* = 2 produced a relatively higher FE current density than did *R* = 3. In addition, when the ratio *R* was fixed, the hexagonal arrangement, at a same applied electric field, exhibited a higher FE current density than did the square arrangement. According to the geometrical arrangement, the hexagonal arrangement had a higher number density than did the square arrangement; this phenomenon was consistent with our results when the amount of emission sites was low (less than 10^7^ cm^−2^).

**Table 2:** Turn-on electric field, enhancement factor, and number density of the samples

Sample	*E*_to_	Enhancement factor	Number density
	(V μm^−1^)	(*β*)	(cm^−2^)
A	2.0	1020	1.0 × 10^5^
B	2.8	840	4.9 × 10^4^
C	1.6	1770	1.6 × 10^5^
D	2.5	905	7.0 × 10^4^

**Fig. 4:**
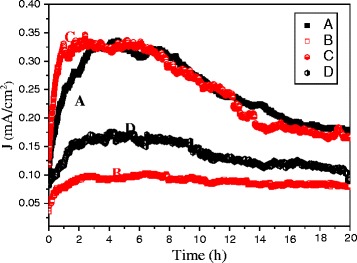
Stability test for samples *A*, *B*, *C*, and *D*

In addition to *E*_to_, which is used to evaluate the performance of a field emitter, the durability and stability are major factors used to evaluate the FE characteristics. The applied electric field was fixed at 3.7 V μm^−1^ for 20 h. Figure 4 shows the long-term measurement of the samples. Two groups of the results were determined and distinguished. One group (samples C and A) revealed a relatively higher FE current density with an ascent and fluctuations in the first hours. Subsequently, the FE current density stabilized gradually. At approximately the sixth hour, the FE current density began to attenuate until the long-term measurement ended at approximately 0.17 mA cm^−2^. Sample A demonstrated a tendency similar to that of sample C. Although samples B and D showed fluctuations during the long-term measurements, the fluctuation extent was less than that of samples C and A. The FE current density of sample D increased in the first 2 h and then stabilized at approximately 0.17 mA cm^−2^. The current decreased gradually from the sixth hour and then became stable at an FE current density of approximately 0.12 mA cm^−2^. Sample B demonstrated the most stable FE current density among the four samples. A similar current density rise was detected in the first 2 h. The FE current density was then maintained at approximately 0.09 mA cm^−2^. A slight drop in current density occurred at the seventh hour, and the current density degraded slightly until the end of the long-term measurement. In other words, the samples with *R* = 2 (samples A and C) reached relatively higher FE current densities, but had acuter fluctuations, whereas the samples with the *R* = 3 (samples B and D) exhibited relatively more stable FE current densities. The occurrence of the fluctuations may have resulted from the heat and mechanical stress from Joule heating during the electric field application [16]. In this study, samples A and C reached a higher FE current density and thus generated more Joule heating, overheating the emitters in a unit area and inducing avalanche degradation.

**Fig. 5:**
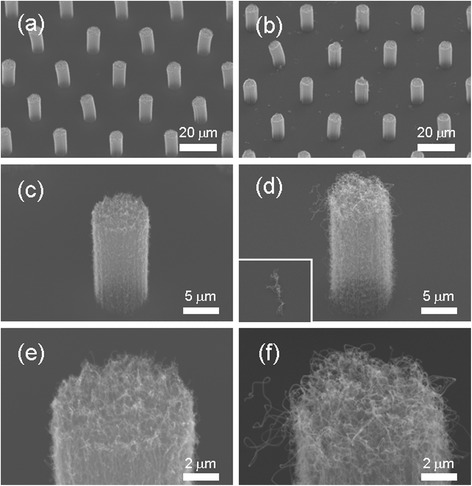
SEM images of the samples before and after long-term measurements. Images at the *left* (**a**, **c**, and **e**) are the samples before measurement and those to the *right* (**b**, **d**, and **f**) are the samples after measurement

To determine the results of the long-term measurement, we used the SEM images to compare the surface morphologies before and after the long-term measurements were performed. Figure 5 shows the SEM images of sample A before (left) and after (right) the long-term measurement. By comparing Fig. 5a, b, we observed that the surface morphology of sample A remained the same. Furthermore, the top of the CNT bundle (Fig. 5d) after the long-term measurement was more sprawling than that of the grown bundle (Fig. 5c). Some reports have indicated that the bombardment of ionic residual gas, generated by emitted electrons on CNTs, might damage the structure of CNTs. The strong electrostatic force exerted on the CNTs during the FE measurement can peel off CNTs from the substrate, causing FE current decay and arcing [17].

**Fig. 6:**
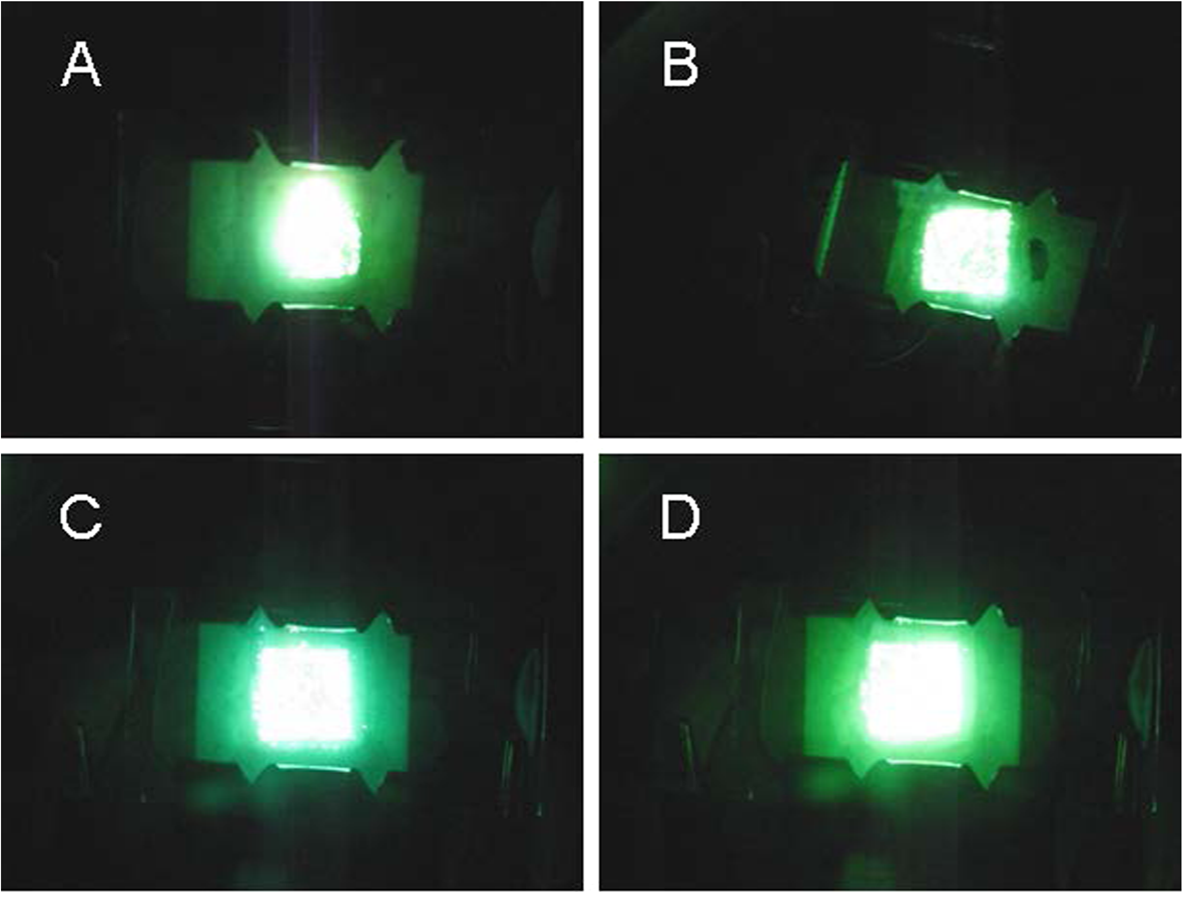
Fluorescence of **a** sample A, **b** sample B, **c** sample C, and **d** sample D

Figure 6 shows fluorescent images of the synthesized samples. The applied electric field was increased gradually until the sample became fluorescent. All the samples with different arrangements and *R*_s_ achieved a uniform fluorescent emission at approximately 4 V μm^−1^. Despite the different FE characteristics of the *E*_to_ and durability, FE uniformity was obtained because of the regular arrangement.

## Conclusions

We used photolithography to successfully define and pattern square- and hexagonal-structured VACNT bundles. Adjustment of the growth time enabled the height of a VACNT bundle to be controlled for *R* = 2 or 3. The hexagonal arrangement with *R* = 2 had the lowest *E*_to_ and the highest *β*, whereas the square arrangement with *R* = 3 had the most stable FE characteristics. Because of the square and hexagonal arrangements, all the samples exhibited FE uniformity. The experimental results of our study demonstrated the practicability of using the VACNT field emitter arrangement to achieve optimal FE performance.

## Abbreviations

CNT: carbon nanotube
CVD: chemical vapor deposition
FE: field emission
FN: Fowler–Nordheim
SEM: scanning electron microscope
VACNTs: vertically aligned CNTs
